# Human plague: An old scourge that needs new answers

**DOI:** 10.1371/journal.pntd.0008251

**Published:** 2020-08-27

**Authors:** Xavier Vallès, Nils Chr. Stenseth, Christian Demeure, Peter Horby, Paul S. Mead, Oswaldo Cabanillas, Mahery Ratsitorahina, Minoarisoa Rajerison, Voahangy Andrianaivoarimanana, Beza Ramasindrazana, Javier Pizarro-Cerda, Holger C. Scholz, Romain Girod, B. Joseph Hinnebusch, Ines Vigan-Womas, Arnaud Fontanet, David M. Wagner, Sandra Telfer, Yazdan Yazdanpanah, Pablo Tortosa, Guia Carrara, Jane Deuve, Steven R. Belmain, Eric D’Ortenzio, Laurence Baril

**Affiliations:** 1 Epidemiology and Clinical Research Unit, Institut Pasteur de Madagascar, Antananarivo, Madagascar; 2 Centre for Ecological and Evolutionary Synthesis (CEES), Department of Biosciences, University of Oslo, Oslo, Norway; 3 Key Laboratory for Earth System Modelling, Department of Earth System Science, Tsinghua University, Beijing, China; 4 Yersinia Research Unit, National Reference Centre “Plague & Other Yersinioses,” WHO Collaborating Research and Reference Centre for Yersinia, Institut Pasteur, Paris, France; 5 Centre for Tropical Medicine and Global Health, Nuffield Department of Medicine, University of Oxford, Oxford, United Kingdom; 6 Bacterial Diseases Branch, Division of Vector Borne Diseases, Centers for Disease Control and Prevention, Fort Collins, Colorado, United States of America; 7 Control de Epidemia Desastres y Otras Emergencias Sanitarias, Oficina General de Epidemiologia, Ministerio de Salud, Perúu; 8 Plague Unit, Central Laboratory for Plague, Institut Pasteur de Madagascar, Antananarivo, Madagascar; 9 Reference Laboratory for Plague, Bundeswehr Institute of Microbiology, Munich, Germany; 10 Medical Entomology Unit, Institut Pasteur de Madagascar, Antananarivo, Madagascar; 11 Rocky Mountain Laboratories, National Institute of Health, National Institutes of Allergy and Infectious Diseases, Hamilton, Montana, United States of America; 12 Immunology of Infectious Diseases Unit, Institut Pasteur de Madagascar, Antananarivo, Madagascar; 13 Emerging Diseases Epidemiology Unit, Institut Pasteur, Paris, France; 14 PACRI unit, Conservatoire National des Arts et Métiers, Paris, France; 15 The Pathogen and Microbiome Institute, Northern Arizona University, Flagstaff, Arizona, United States of America; 16 School of Biological Sciences, University of Aberdeen, Aberdeen, United Kingdom; 17 REACTing, Inserm, Université Paris-Diderot, Sorbonne Paris Cité, Paris, France; 18 Service de Maladies Infectieuses et Tropicales, Hôpital Bichat-Claude Bernard, AP-HP, Paris, France; 19 Université de La Réunion, Unité Mixte de Recherche Processus Infectieux en Milieu Insulaire Tropical, La Réunion, France; 20 Department of International Affairs, Institut Pasteur, Paris, France; 21 Natural Resources Institute, University of Greenwich, Chatham Maritime, Kent, United Kingdom; Beijing Institute of Microbiology and Epidemiology, CHINA

## Abstract

*Yersinia pestis*, the bacterial causative agent of plague, remains an important threat to human health. Plague is a rodent-borne disease that has historically shown an outstanding ability to colonize and persist across different species, habitats, and environments while provoking sporadic cases, outbreaks, and deadly global epidemics among humans. Between September and November 2017, an outbreak of urban pneumonic plague was declared in Madagascar, which refocused the attention of the scientific community on this ancient human scourge. Given recent trends and plague’s resilience to control in the wild, its high fatality rate in humans without early treatment, and its capacity to disrupt social and healthcare systems, human plague should be considered as a neglected threat. A workshop was held in Paris in July 2018 to review current knowledge about plague and to identify the scientific research priorities to eradicate plague as a human threat. It was concluded that an urgent commitment is needed to develop and fund a strong research agenda aiming to fill the current knowledge gaps structured around 4 main axes: (i) an improved understanding of the ecological interactions among the reservoir, vector, pathogen, and environment; (ii) human and societal responses; (iii) improved diagnostic tools and case management; and (iv) vaccine development. These axes should be cross-cutting, translational, and focused on delivering context-specific strategies. Results of this research should feed a global control and prevention strategy within a “One Health” approach.

## Author summary

The historical aspect of plague makes for fascinating reading, due to its capacity to disrupt human society and its socioeconomic and cultural impacts throughout human history. We argue that the Madagascar outbreak in 2017 is a tipping point in human plague epidemiology and a call to elevate research priorities on plague as a matter of some urgency. In contrast with what occurred with the Ebola virus disease crisis in West Africa between 2013 and 2015 and the new coronaviruses (the emergence of severe acute respiratory syndrome coronavirus [SARS-CoV] and Middle East respiratory syndrome coronavirus [MERS-CoV] as early warnings of the current severe acute respiratory syndrome coronavirus 2 [SARS-CoV-2] pandemic), we have an opportunity to act preventively and enable evidence-based measures to avoid major health crises due to plague outbreaks in the near future.

## Introduction

**Fig 1:**
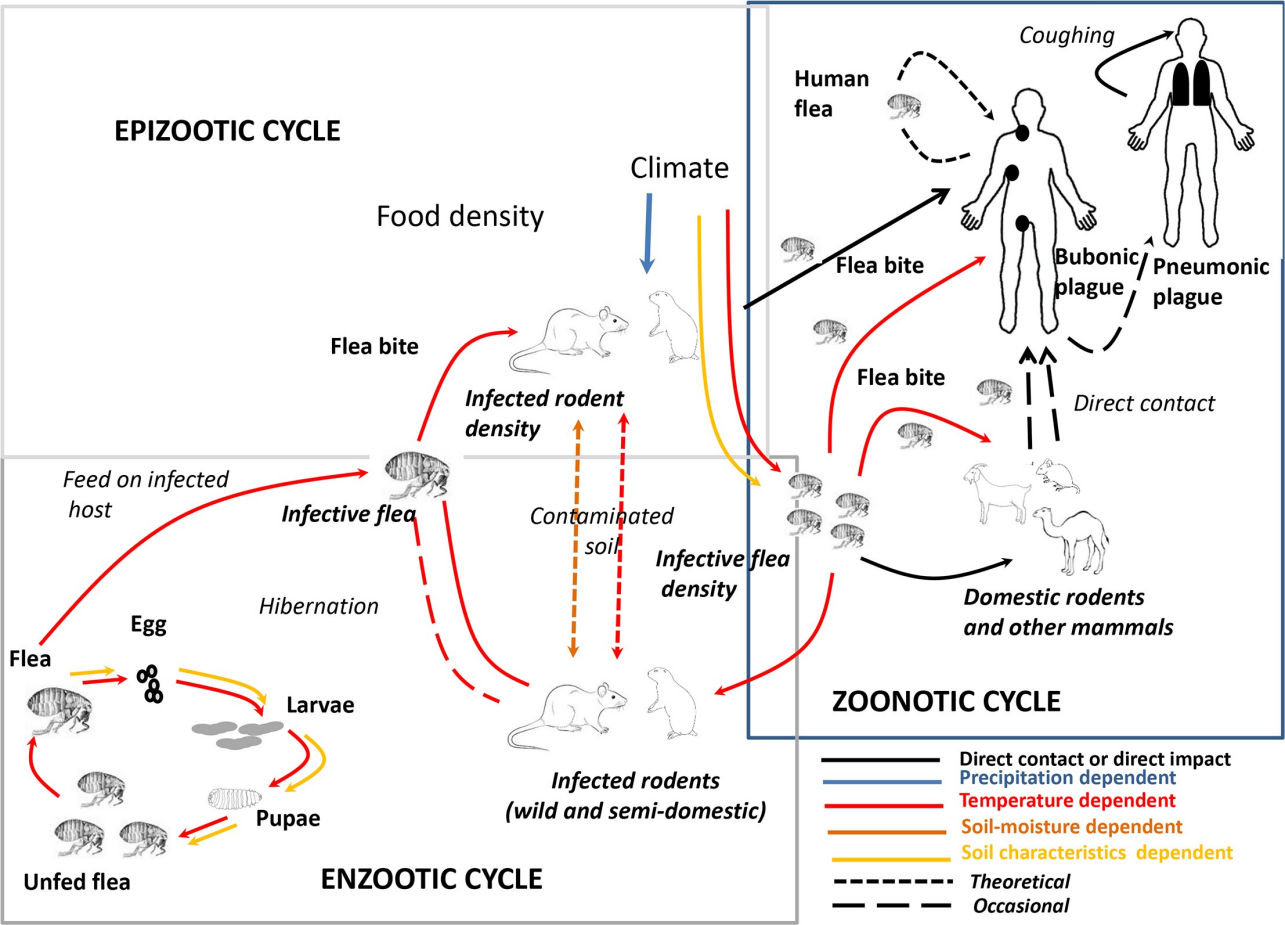
Epizootic/enzootic cycle of *Y*. *pestis*. Caption credit: Ben Ari MT, Neerinckx S, Gage KL, Kreppel K, Laudisoit A, Leirs H, et al. Plague and Climate: Scales Matter. PLoS Pathog. 2011; 7:e1002160 [49].

Plague is a bacterial rodent-borne disease caused by *Yersinia pestis*, a gram-negative bacillus member of the Enterobacteriaceae family. As a zoonosis, it is first and foremost a rodent disease with complex zoonotic/epizootic cycles that may occasionally be transmitted to humans, whereby it can cause sporadic cases, outbreaks, or even large epidemics (Fig 1). Bubonic plague is the most common clinical presentation among humans. Bubonic forms may evolve to septicaemic disease, and 1% to 3% of cases develop a secondary pneumonic plague [1–3]. Secondary pneumonic plague forms may be transmitted from person to person through respiratory droplets, which can result in primary pneumonic plague. Without intensive treatment, the lethality of septicaemic and pneumonic plague is almost 100% between 1 and 4 days following the onset of symptoms [1–3].

Plague has marked human history in a unique way during at least 3 historical pandemics. The first described pandemic was the Justinian epidemic (6th–7th centuries), whereas the second spanned from the 14th century to the 19th century in Europe [4, 5], including the Black Death period (1,347–1,351 BC) that wiped out an estimated 30% to 40% of the European population, constituting its deadliest recorded epidemic. The third pandemic of plague emerged from its natural cradle in the Yunnan province (China) in the mid-19th century [6]. Because of the expansion of the shipping industry, this third wave established sustained *Y*. *pestis* epizootic cycles worldwide, including in the United States, South America, Madagascar, and other areas previously free of plague. Plague is currently endemic in restricted areas where it has been present for several hundred or even thousands of years (China, Kyrgyzstan, Kazakhstan, Russia, and Mongolia), or just a hundred years (Peru, the US, Madagascar, and some areas in Africa) [5, 7, 8]. Although in the mid-20th century human cases were mostly reported from Asia, with a steadily decreasing trend, a sharp increase of cases in Africa was observed during the 1980s to the 2000s, which now make up the majority of total cases [4]. Between 2013 and 2018, 2,886 cases and 504 deaths have been notified to WHO (with a reported case fatality of 17.5%), of which 95% derive from sub-Saharan Africa, mainly in Madagascar and the Ituri region of the Democratic Republic of Congo (DRC) [8]. Occasional scattered cases of human plague are regularly declared in China, Mongolia, the Russian Federation, Kyrgyzstan, Peru, Bolivia, Uganda, Tanzania, and the US [7–9]. Nowadays, *Y*. *pestis* remains present in at least 33 countries (where signs of activity have been detected in the last 30 years). More than 30 different flea vectors have been suspected to play a role in transmission, and over 200 mammals (mainly rodents and lagomorphs) have been reported to be infected by *Y*. *pestis*, including potential reservoir hosts, in different parts of the world. Table 1 exemplifies this diversity. However, the commensal rats (*Rattus rattus* and *R*. *norvegicus*) and their flea (*Xenopsylla cheopis*) are currently considered the most important hosts and vector involved in human outbreaks. Other hosts and vectors are involved in human transmission in different regions. Overall, plague is largely a disease of wild mammals, killing susceptible rodent species, cats, camels, and other mammals when the disease spills over from rodent reservoir host species. The maintenance of *Y*. *pestis* in nature, and from where it spreads to cause human outbreaks, renders it almost impossible to eradicate.

**Table 1:** Main vectors and hosts identified in countries where human plague has been declared between 2013 and 2018*.

Country	Declared human plague cases 2013–2018**	Recognized main host(s)	Described vectors involved in zoonotic/epizootic cycles
DRC [7]	410	*Arvicanthis abyssinicus* *Mastomys natalensis* *Lemniscomys striatus* *Rattus rattus* *Mus minutoides*	*Dinopsyllus lypusus* *Ctenophthalmus cabirus* *Ctenophthalmus phyris* *Xenopsylla brasiliensis*
Madagascar [7,100,108]	2,323	*R*. *rattus* *R*. *norvegicus* *Suncus murinus*	*X*. *cheopis* *X*. *brasiliensis* *Synopsyllus fonquerniei*
Uganda [7,56]	22	*Arvicanthis niloticus* *Mastomys *spp. *Crocidura *spp.	*X*. *cheopis* *X*. *brasiliensis* *C*. *cabirus* *D*. *lypusus*
United Republic of Tanzania [7]	36	*R*. *rattus* *M*. *natalensis* *A*. *abyssinicus*	*X*. *cheopis* *X*. *brasilensis* *D*. *lypusus*
Bolivia [7,98]	3	*R*. *rattus* *Graomys griseoflavus* *Galea musteloides*	*X*. *cheopis*
Peru [98]	40	*R*. *rattus* *R*. *norvegicus* *Sciurus stramineus* *Akodon mollis* *Cavia porcellus* *Aegialomys xanthaeolus* *Oryzomys andinus*	* * *Polygenis litargus* *X*. *cheopis* *Hectopsylla* spp. *Tiamastus cavicola* * *
US [25, 109]	40	*Cynomys gunnisoni* *Cynomys ludovicianus* *Onychomys leucogaster* *Otospermophilus variegatus* *Otospermophilus beecheyi* *Callospermophilus lateralis* *Urocitellus beldingi* *Eutamias* spp. *Microtus californicus*	*Oropsylla hirsuta* *Oropsylla montana* *Opisocrostis spp*. *Diamanus montanus* *Hoplopsyllus anomalus*
China [6,7]	5	*Marmota himalayana* *Marmota caudata* *Rattus flavipectus* *Urocitellus undulatus* *Spermophilus dauricus* *Eothenomys miletus* *Apodemus chevrieri* *Meriones unguiculatus* *Microtus brandti* *Microtus fustus*	*Callopsylla dolabris* *Oropsylla silantiewi*
Kyrgyzstan [6,110]	1	*Marmota baibacina* *Microtus gregalis* *Microtus carruthersi* *M*. *caudata*	*Callopsylla caspia* *O*. *silantiewi* *Citellophyllus tesquorom*
Russia Federation [110]	1	*Spermophilus pygmaeus* *Meriones meridianus* *Ochotona pallasi pricei* *U*. *undulatus* *S*. *dauricus* *Spermophilus musicus* *Microtus arvalis*	*C*. *caspia* *Neopsylla setosa* *Neopsylla laeviceps* *X*. *conformis* *Paradoxopsyllus scorodumovi*
Mongolia [7,110–113]	5	*Marmota sibirica* *Rhombomys opimus* *M*. *unguiculatus* *Allactaga sibirica* *Cardiocranius paradoxus*	*O*. *silantiewi* * *

*A comprehensive review of hosts and vectors involved or suspected to be involved in plague transmission would need an entire article or chapter of a book. The aim of this table is to highlight the outstanding ability of *Y*. *pestis* to evolve in different hosts and vectors.

**From: Bertherat E. Plague around the world in 2019. Weekly Epidemiological Report. 2019;25:289–292 [8].

**Abbreviation:** DRC, Democratic Republic of Congo

Plague once again grasped the attention of the scientific community when an outbreak of pneumonic plague was declared in Madagascar in September 2017, primarily striking the capital Antananarivo and the main seaport of Toamasina. A total of 1,878 clinically suspected pneumonic plague cases were identified [10]. This episode should be considered an echo of the third pandemic, which first established plague in Madagascar [11].

Pneumonic plague outbreaks with human-to-human transmission are fortunately relatively rare, probably due to the estimated low basic reproduction number (R_0_) between 1.2 and 1.4 [12, 13]. Besides the recent outbreak in Madagascar, minor episodes of pneumonic plague have been described in recent years in the US [13], Madagascar [14, 15], India [16], DRC [17,18], Uganda [19], Peru [20], and China [21]. However, large and deadly outbreaks of pneumonic plague have occurred, most notably in Manchuria, where more than 60,000 estimated deaths occurred between 1910 and 1911 [3, 22], highlighting that R_0_ of pneumonic plague depends on the specific situation and interaction of vulnerable population as has been showed during the analysis of more recent outbreaks [15] and past outbreaks [3]. Therefore, given the high lethality, the capacity for social disruption, increasing connectivity between endemic and rural areas, and international transport, plague should be considered a neglected threat that needs renewed attention.

This article is based on the conclusions of a workshop held by international plague experts in Paris in July 2018 that had the main goal of drafting a roadmap on plague research priorities, expanding the scope and contents of a short report published elsewhere [23]. With this purpose, we summarize the current knowledge about plague and identify the priority gaps to be filled based on the exchange of knowledge and experiences during the meeting and a systematic review of published plague literature. Here, we report the main conclusions of the discussions at this meeting.

## Which hosts and vectors should be targeted for human plague control?

It is important to point out that the true number of species implicated in plague transmission—the true composition of reservoirs and accidental hosts—is unknown [24]. Therefore, one major challenge in the management and prevention of plague is to identify the flea vector(s) and small mammal reservoir(s) in different parts of the world where plague has long been established. In most plague foci, several vectors and reservoirs are implicated in plague transmission and persistence; however, in some plague foci, there is limited research and only partial knowledge of the vector and reservoir species involved. Different vectors and hosts (including intermediary hosts between wild and domestic animals) have been described in disparate settings and ecological conditions: from equatorial forests in the DRC to arid regions in central Asia. Since the management priority has normally been to control human plague by preventing transmission to humans, researchers have tended to focus on the interface between humans and flea vectors, while the interactions between sylvatic and commensal small mammals, vectors, and landscape ecology have received relatively little attention. Factors responsible for epizootic spread (episodic amplification within and between mammal species) vary in different settings, and reservoirs may be multiple or unique. Domestic mammals and some atypical vectors could play a bridge role between wild hosts and the human environment. In the US, the increase of intermediary hosts populations of the grasshopper mouse (*Onychomys leucogaster*) is thought to enhance the connectivity between primary hosts populations of prairie dogs (*Cynomys ludovicianus*), the risk of plague outbreaks in the wild [25], and in turn the chances of human transmission. Although fleas have been assumed to constitute the main drivers of host-to-human transmission, this may depend on different contexts. In Madagascar, *X*. *cheopis* is predominantly found on black rats living in houses, while *Synopsyllus fonquerniei* is found on the fur or within burrows of black rats living outside houses but also in open biotopes and forests, where it may parasitize endemic insectivores and rodents [26], with a synergistic role on plague persistence [27]. Similar situations may be found across other plague-endemic areas, thus highlighting the diversity of hosts and vectors as a key factor for plague persistence in the wild. In fact, *Y*. *pestis* has been detected in a large number of fleas (Table 1) and other ectoparasites; their roles throughout plague-endemic regions of the world remain unclear. A recent study suggests that inter-human transmission through ectoparasites (*Pulex irritans* and *Pediculus humanus*) may have played a predominant role during the historical Black Death Pandemic [28]. In fact, both of these ectoparasites are capable of carrying *Y*. *pestis* [29, 30]. Furthermore, it has been pointed out that specific so-called vectors are acting as true hosts, since they are able to carry *Y*. *pestis* for weeks.

Current data indicate that host and vector population structures and hotspots of plague have a crucial influence on plague epidemiology. It has been suggested that the balance between resistant hosts (those able to harbour *Y*. *pestis* with no apparent ill effect) and susceptible host (those able to tolerate *Y*. *pestis* for only short periods and eventually succumb to the effects of the bacteria) is important for plague persistence in the wild or resurgence among humans, and susceptible hosts [24]. For instance, modelling and empirical data from Madagascar indicate that rat genetic population structure (the balance between susceptible and resistant hosts) plays a crucial role [31–35] as modelled by topography (see below). This model may also be applied between different species, including multi-host and intra-host interactions. Finally, the implication of amoebae as biotic reservoirs has recently received some experimental and field-based support and therefore deserves consideration [36].

The persistence of plague in the wild or the onset of plague among humans may be related to the internal dynamics of enzootic (maintenance in the wild)/epizootic cycles. This has been well documented in Kazakhstan, where threshold densities of great gerbils (*Rhombomys opimus*) and fleas are correlated with persistence of plague in the wild or resurgence of plague in humans, respectively [37, 38]. Empirical observations elsewhere corroborate this complex dynamic, which should in turn help us to understand the ability of *Y*. *pestis* to regularly emerge in areas where plague has been silent for years. A further question to explore is the capacity of *Y*. *pestis* to survive in soil or post-mortem tissues and contribute to further transmission, as a potential link to enzootic and even epizootic cycles [39, 40].

The understanding of the biology of host-vector-pathogen interaction and proximity to humans would aid development of specific control strategies, and to address apparently simple but not well-answered questions, such as the relative efficacy of using domestic flea control on its own or in combination with rodent control or reservoir host vaccination. Mechanisms of host and vector control more commonly involve the combined use of flea and rodent control, or community-based interventions such as encouraging behavioural changes or environmental interventions (e.g., improved hygiene and sanitation). The use of chemicals to eliminate fleas or rodents requires the surveillance of physiological resistance mechanisms in flea/rodent populations and the identification of biochemical or genetic markers of resistance as well as the characterization of mechanisms involved and their environmental and genetic determinants. Studies on *X*. *cheopis* insecticide resistance have already been carried out in different situations [41, 42]. However, information on other flea species is most often missing. Beyond insecticides, rodenticides, and the problem of resistance, innovative tools and methods for flea and rodent control must be proposed, tested, and validated in the field, including nontoxic solutions and strategies (i.e., physical methods like cellulose glue-based products for flea control).

The implementation of rodent or vector control measures should also be carefully evaluated, given the complexity of rodent/vector interactions in different settings. For instance, experience in Peru has suggested that reducing the population of particular rodent species may in turn act as a risk factor for increasing intra- and interspecific contact rates among rodent species and with the human population, leading to a resurgence in human plague cases. Similar observations have been made with other rodent/mammal-borne diseases, such as leptospirosis [43] and bovine tuberculosis [44], whereby host control (rats and badgers, respectively) can lead to increased movement by those animals remaining, facilitating disease spread and higher disease prevalence. This strongly suggests that applying universal measures may be counterproductive in different scenarios.

Besides the use of insecticides or rodenticides, there are specific human behaviours that could be modified to avoid proliferation of domestic hosts or the chances of contact with infected vectors. Some behavioural factors that favour the transmission of *Y*. *pestis* to humans are well known in on-going plague surveillance programmes. In Peru, plague cases have been correlated to human activities, e.g., domestic breeding of guinea pigs within people’s homes, the storage of harvest in unsealed conditions, and the poor hygienic living conditions of temporary workers [45]. In Madagascar, a close link exists between seasonality of human plague and the movement of rats between houses and rice fields related to the harvesting season and the use of slash-and-burn cultivation [26, 46]. During the 2004 outbreak in DRC [47], inter-human pneumonic plague transmission could be favoured by the housing conditions of gold and diamond mine workers.

## What are the drivers of human plague?

Drivers of human plague include contextual factors that influence host and vector population dynamics and human behaviours that may increase the chances of exposure and infection. Such factors mainly include altitude, temperature, rainfall, biome, geography, and anthropogenic environmental changes through deforestation, agricultural expansion, cropping systems, activities and patterns, climate change, urbanisation, and the introduction of non-native invasive species.

For instance, in the DRC an increase of plague was observed after the introduction of *Rattus* spp. into the wild combined with the introduction of new crops to replace cattle farming [47]. In Madagascar, *R*. *rattus* was introduced centuries before the arrival of *Y*. *pestis*, most probably through the Arabian trade network in the Indian Ocean, which was flourishing from the middle of the first millennium [48]. Climate is considered to have a major impact on plague incidence [49]. Rainfall and temperature have correlated with the increase or decrease of gerbils or vector populations in Kazakhstan [37], and rainfall was linked to human plague occurrence in Uganda [50], which, in turn, strongly influences the abundance of small mammals [51], thus increasing the probability of epizootic cycles. Studies in the US have shown relationships with *El Niño* episodes and the effects of rainfall on host populations and temperature on vector survival [52, 53]. Similarly, *El Niño* episodes have been considered an early warning in Peru [45] and Madagascar [54]. Temperature anomalies and altitude have been related to plague occurrence in Madagascar [55]. Temperature and humidity influence the differential development and survival of the 2 main flea vectors (*X*. *cheopis* and *S*. *fonquerniei*) involved in plague transmission in Madagascar (where plague shows a clear seasonal pattern) [27]. There is a clear interplay between plague occurrence, human behaviours, climate, and landscape in a number of contexts [56–59]. Data from Madagascar suggest that geography shaped the genetic diversity of hosts (rats) and *Y*. *pestis* as a result of the relative population isolation, which may play an important role for *Y*. *pestis* persistence, through the reintroduction of new strains of hosts and/or the pathogen between different areas [60–62]. Furthermore, geography and landscape may influence the risk and directionality of plague spread through their hosts [63]. The confirmation of the drivers of plague should help to better map the risk of plague, to improve surveillance, preparedness, and focused interventions.

The connection between rural and urban areas is a major concern to avoid urban outbreaks. The reduction or discontinuation of surveillance programmes and the exacerbation of poverty and poor sanitation are important factors in the emergence of human cases. The 2017 outbreak in Madagascar raises the question of whether specific determinants exist for bubonic or pneumonic outbreaks: climatic oscillations with earlier seasonal slash-and-burn farming practices, a lack of vigilance from the health authorities leading to the absence of efficient communication to the population and lower healthcare standards and surveillance systems. However, only human population density has been consistently related to the risks of human to human transmission of pneumonic plague. No specific strains of *Y*. *pestis* have been associated with particular virulence factors or tropism for pneumonic forms.

## Which new diagnostic tools for plague are needed?

The WHO gold standard for plague diagnosis remains the bacterial culture. However, culture is not feasible in most of the settings in which plague occurs as proper handling of samples is sometimes difficult, and the results are not immediately available (minimum 4 days). In addition, isolation of *Y*. *pestis* is hampered by administration of antibiotics prior to sampling. Therefore, case management is essentially based upon symptomatic diagnosis, which is specific to endemic areas with bubonic forms. Pneumonic cases pose particular diagnostic challenges, since it is rarely suspected during the initial stages of an outbreak. Initial symptoms of pneumonic plague without an evident bubo (lymph node swelling) are nonspecific. Collection of high-quality sputum from suspected pneumonic plague patients is particularly difficult to obtain, more so than aspirates of bubo, and even if good quality sputum is obtained, the processing stage of this material, polymicrobial, and complex specimen is technically problematic. As a result, the yield from sputum examination and cultivation is very low.

The rapid diagnostic test (RDT) as point of care (POC) strategy was implemented in Madagascar in 2002 in primary care facilities based in the “endemic districts” together with information provided to community health workers. This RDT has been widely used and improved the specificity of diagnosis, resulting in a drop in the number of suspected cases of bubonic plague [64]. The RDT is based on monoclonal antibodies against the F1 capsular antigen of *Y*. *pestis*. F1 detection in samples collected directly from bubonic aspirate are processed and interpreted by the healthcare workers. The combination of bacteriological methods and F1 ELISA have positive and negative predictive values of 90.6% and 86.7%, respectively [65]. However, this RDT was evaluated during the Madagascar pneumonic plague outbreak of 2017, and the sensitivity and specificity for sputum samples was limited; further analysis is on-going. Conventional PCR targeting *pla* [66, 67] and *caf1* genes [68, 69] has also been used for investigations in the past. In the context of the Madagascar outbreak, a new algorithm based on real time (targeting *pla*, *caf1* genes) and conventional PCR (targeting *inv*_*1100*,_ *caf1* and *pla*) has been implemented to ensure a rapid and reliable diagnostic [10]. Serology based on anti-F1 capsular antigen-specific immunoglobulins (IgG/IgM) [70] is used for prevalence surveys among humans, mammals, and post-outbreak investigations, but it may have questionable value in pneumonic plague. Therefore, rapid and reliable diagnostic tests are necessary for both bubonic and pneumonic plague for an efficient outbreak response.

Different plague diagnostic options have been proposed, from improvement of culture methods to appropriate molecular methodologies. Proposed methods are summarized in Box 1.

**Box 1. Proposed improved plague diagnostic methods**

1. **Improve the yield of *Y*. *pestis* culture**
  - *Better sample collection*, *transport*, *and processing (homogenization)*
  - Developing more selective media for cultivation from sputum and bubo aspirates
2. **To improve the sensitivity and specificity of RDT**
  - More adequate protocols for more efficient F1 antigen release
  - To develop a second generation of RDT through the inclusion of additional antigens
3. **New nucleic acid–based tools**
  - Quantification of DNA in sputum samples
  - PCR-based detection in blood
  - PCR for saliva
4. **Upgrade serological testing methods through the inclusion of more antigens and/or alternative to current ELISA methods**

In addition, especially for pneumonic plague, capabilities for differential diagnostic should be improved, and the use of new techniques such as metagenomics in sputum could help to estimate the true magnitude of an outbreak. The use of these diagnostic tools among animals (domestic or wild) as sentinel surveillance mechanisms should also be considered.

## How can plague surveillance and case management be improved?

Surveillance of plague is of utmost importance in endemic regions, first for early case detection and treatment (which is related to favourable outcome); second, for early warning of human plague occurrence or outbreaks; and, third, for more accurate quantification of the disease burden and geographical distribution. Most human plague cases are currently occurring in underpopulated and remote areas with particularly difficult access. True case numbers of human plague may be underestimated in some areas, especially in areas with weak health systems and unstable social and political situations, like in the DRC. Therefore, the accuracy of current surveillance systems for human plague cases is still uncertain, to which is added the existence of pauci-symptomatic or asymptomatic cases suggested by some sero-surveillance studies [71]. However, plague surveillance should be integrated into regular surveillance systems of common diseases in impoverished regions, and therefore general improvement of the Health Information System is needed, since plague is not usually the leading health issue.

Plague surveillance should integrate human, climate, and animal surveillances as well as a combination of passive or active case detection depending on the scenarios, the season, or the early warning signs. A central question regarding surveillance is identifying which reservoir hosts and vectors are the most appropriate for sentinel surveillance. Once again, the answer may be different in different settings. Classical pre-outbreak deaths among hosts (rats or other rodents), which typically indicates an explosive spread of an epizootic cycle, is not systematically observed and may even be an exception perpetuated by classical accounts [72]. Experiences in Peru pointed out the utility of some domestic animals, like dogs or cats, as sentinel indicators of an increased risk of infection. Some studies found a predictive plague occurrence value of seroprevalence among certain mammals [73, 74].

Cases may arise in a community with a high degree of stigma linked to plague and therefore be hidden from local authorities. Changing deeply rooted cultural behaviours may be difficult and increase the risk of outbreak by delaying health care-seeking behaviours and making access to healthcare centres difficult. Community-based surveillance systems by community health workers or networks of community-based key informants have been used to increase awareness and as early warning systems for different diseases, including plague in Madagascar. As with other infectious diseases, when cases arise in areas or countries where plague is not usually reported or with uncommon clinical forms (i.e., pneumonic plague), a lack of suspicion may lead to a critical delay in notification, as it was reported during the early stages of the 2017 outbreak in Madagascar.

Early detection, diagnosis, and treatment are crucial for a favourable outcome and depend on an efficient surveillance system. A number of treatment protocols have been used to treat plague (Table 2); however, only a small randomized trial including gentamycin and doxycycline has been conducted [75]. Different therapeutic choices have been based on empirical observations or animal models. The most widely recommended treatment is a high dose of streptomycin, resulting in effective clinical responses in both bubonic and pneumonic plague [1, 2]. However, streptomycin has side effects such as deafness and renal toxicity, and the administration to children and pregnant women is problematic. The administration protocol (intramuscular) remains a logistical and material challenge in resource-poor settings, as well as in the context of an outbreak. In addition, streptomycin is often not available as it is no longer used as a first-line treatment of tuberculosis. As shown during the recent plague outbreak in Madagascar, the lack of specific clinical and laboratory diagnostics for pneumonic plague was responsible for an important number of misdiagnoses leading to an overestimate of the magnitude of the outbreak [10] and, consequently, inadequate treatment of severe community-acquired respiratory infections with streptomycin. The situation was worsened by the lack of resources to carry out rapid and specific exclusion diagnostics for other pathogens. During an investigation of pneumonic plague in Ituri, DRC, it was found that leptospirosis cases were included as suspected plague cases [76]. On the other hand, healthcare providers should be trained to provide chemoprophylaxis to decrease secondary cases and should be equipped to avoid nosocomial infections.

**Table 2:** Current treatment protocol (WHO and CDC recommendations)*.

Antibiotic	Dose	Duration	Remarks	Way of administration
Streptomycin	15 mg/kg/dose	7 days	Maximum 1 g per day	IM
Gentamycin	5 mg/kg/d	7 days	Once daily	IM or IV
Doxycycline	100 mg/12 h	7 to 10 days	Initially a loading dose of 200 mg/12 h the first day	IV or PO
Tetracycline	2 g/d	7 to 10 days	Initially loading dose of 2 g	IV or PO
Levofloxacin	500 to 750 mg daily	7 to 10 days	Once-daily special indication for high tissue penetration	IV or PO
Ciprofloxacin	400 mg/12 h	7 to 10 days		PO
Chloramphenicol	50 to 100 mg/kg/d	10 days	Indicated in cases for high tissue penetration	IV or PO
			Dose may be reduced to 25 to 30 mg/kg/d depending on clinical response to reduce the risk of bone marrow suppression	
Cotrimoxazole	1 g/4–6 h	10 days	Not indicated as first choice	PO
			Initial loading dose of 2–4 g	
**Approved combinations**				
Gentamycin + doxycycline	*See above*			
Gentamycin + levofloxacin	*See above*			
**Postexposure prophylaxis**				
Doxycycline	100 mg/12 h			PO
Cotrimoxazole	ND			
Ciprofloxacin	500 mg/12 h			

Modified from: Mead PS. Yersinia Species (Including Plague). In: Mandell, Douglas, and Bennett's. Principles and practice of infectious diseases, 8th Edition; 2015. pp. 2607–2618 [2].

**Abbreviations:** CDC, Centers for Disease Control and Prevention; IM, intramuscular; IV, intravenous; ND, not defined; PO, by mouth

These experiences indicate that validation of therapeutic alternatives are needed. A number of different alternatives to streptomycin have been empirically suggested (see Table 2). Clinical trials need to be set up during outbreaks to evaluate these therapeutic alternatives. New guidelines from the Ministry of Public Health of Madagascar are now recommending the combination of levofloxacin and gentamycin for hospitalized pulmonary cases. They also give the possibility to switch to other treatments after the acute phase (switch to ciprofloxacin after the early phase of streptomycin treated cases). Gentamycin, fluoroquinolones, chloramphenicol, doxycycline, and trimethoprim-sulfamethoxazole are different alternatives used in different settings, which generally result in good clinical outcomes [1, 7, 77]. A well-established first-line treatment for suspected pneumonic plague needs to be established that is affordable, is orally administered, has few side effects, is continuously available, and has a wide spectrum of activity to target other community-acquired respiratory diseases.

The possibility of *Y*. *pestis* to exchange resistant coding plasmids with other members of the Enterobacteriaceae family, which can easily carry almost any sort of drug resistance, is a serious threat [78]. *Y*. *pestis* has been shown to be sensitive to antibiotics in strains isolated from humans; however, 3 unrelated strains have been isolated from hosts and vectors in Madagascar which carried plasmid transmitted resistance to streptomycin and other drugs [79, 80]. The extreme virulence of *Y*. *pestis* further stresses the need to set up reliable monitoring of susceptibility to treatment.

## What are the gaps in knowledge about *Y*. *pestis* biology?

Although much is known about specific virulence factors of *Y*. *pestis* [81, 82], further technical development regarding specific prevention or therapeutic tools or drugs has not occurred, which would require a deeper understanding of the underlying biology of *Y*. *pestis*. For instance, current data indicate that the degree of blockage of the biofilm in the flea’s gut is vector specific and is responsible for the biofilm-dependent transmission efficiency of infected vectors—or the ability to carry *Y*. *pestis* over days or weeks—and a key step in *Y*. *pestis* transmissibility [83, 84]. Drugs targeting the biofilm formation in the guts of flea vectors could be a promising strategy.

One interesting avenue of research is genetic expression studies of *Y*. *pestis*, which may help to understand pathogen-vector-host interactions and their persistence [85]. An example of this approach is the identification of 10 conserved genes that lead *Y*. *pestis* to survive in soil and post-mortem tissues. This suggests that this pathway plays a relevant role in *Y*. *pestis* persistence [86]. In fact, the study of the interplay of pathogenicity and evolution has given important insights about *Y*. *pestis* biology. The combination of gene acquisition (through horizontal exchange) and loss in natural populations of *Y*. *pestis* explains their outstanding adaptability to different hosts, vectors, and environments [6, 87]. The high virulence of *Y*. *pestis* could be a result of an adaptive response to generate particularly high levels of bacteraemia in order to ensure transmissibility between hosts and vectors [88]. With respect to hosts and vectors, an interesting area to explore is to ascertain the selective process that plague has exerted upon mammal populations [89], including humans [90]. The selection of resistant hosts or intermediate-susceptible hosts in a suitable region is a mechanism of establishment and long-term persistence of *Y*. *pestis*. In turn, this may provide insights on the pathophysiology of plague, through the determination of specific alleles or genotypes, selected by natural pressure of plague which may confer natural resistance. The natural cellular immune response to human pulmonary cases during acute, early, and convalescent phases of the infection in particular is not well understood. Mice models suggest a local immunosuppression effect that may explain the lack of immune response in early stages and the rapid progression of this clinical form [91].

The practical results of this research could be specific drugs that may prevent *Y*. *pestis* spread in the wild or humans, new antibiotics or better shaped control, and prevention measures based on the knowledge of their intimate biological cycle, e.g., the development of more adequate therapies or prophylaxis strategies through the development of vaccines. In this sense, vaccines against *Y*. *pestis* have been developed since the early identification of the pathogen. A live attenuated vaccine (EV76) was developed and used in Madagascar and introduced in Vietnam, Indonesia, and in the former USSR [92]. Due to the significant side effects and the need of revaccination, its use was abandoned once the number of cases fell. Its Russian EV-NIIEG derivative is still used in Asia and Russia [93]. A number of other candidates are under investigation, including whole-cell–based or subunit-based DNA vaccines—attenuated or with live carriers—combining different recombinant antigens and with molecular adjuvants [94]. To date, no phase III clinical trial has been carried out. However, rF1V and SV1 vaccines successfully passed phase II trials; WHO recently provided guidance for phase III evaluation in the field [95]. Given the current extent of human plague, a human vaccine candidate should be effective for immediate distribution in an outbreak context and be effective against pulmonary forms. One promising strategy could be reservoir host vaccination campaigns particularly as vaccination of reservoir hosts is a tried and tested strategy for many zoonotic diseases such as wild foxes for rabies in Europe [96] and livestock for leptospirosis in New Zealand [97].

To sum up, for human plague control, 3 operational targets have been defined for *Y*. *pestis* biology research: (1) identify molecular targets susceptible for drug development in addition to the currently used antibiotics, (2) improve our understanding of the underlying molecular mechanisms of *Y*. *pestis* ability to spread and persist over centuries in different eco-epidemiological settings, and (3) improve our understanding of the immune response to plague that may pave the way for vaccine development and better diagnostic tools.

## Discussion

The horrifying impact of plague during the 14th century is still hard to forget as it is reflected in European folklore, language, and literature. The 2017 Madagascar outbreak should be taken as a serious warning and confirms that plague remains a human threat. However, given this fatal past background and recognized virulence, it is striking to realize that plague is mostly seen as an historical curiosity. It is alarming, indeed, that the most basic tools for control and prevention of plague, such as curative and preventive treatments and vaccines, are still pending adequate validation. This is not related to the lack of robust candidates but to the fact that plague has been a neglected disease during recent decades. Peculiarly, recent plague vaccine development and its funding has long been motivated more by the desire to fight plague as a potential bioterrorism weapon rather than as a public health problem in endemic countries. This is in stark contrast with the accelerated development of Ebola and SARS-CoV-2 vaccines.

Nevertheless, given the diversity of hosts and vectors involved in different parts of the world where plague is endemic, context-specific research is still lacking, particularly in plague-affected low- and middle-income countries. The contrasts in ecological dynamics of vectors and reservoir hosts involved in different locations where plague circulates are remarkable. For instance, in Madagascar, *R*. *rattus* has been recognized as the only relevant host during regular and sustained zoonotic and epizootic cycles, whereas in South America, a surprisingly large range of small mammals has been described to carry *Y*. *pestis* [98]. It is clear that the knowledge of the dynamics of enzootic and epizootic cycles remains fragmented and poorly understood. Indeed, while animal–human transmission is usually investigated following emergence episodes, it is most important to understand the biological cycle of *Y*. *pestis* in the environment in between 2 successive outbreaks. The seasonality of plague in Madagascar makes this environmental setup well-adapted to conduct such research programs.

Plague foci contexts may be rather different between those where the disease occurs or has until recently occurred in a regular and rather predictable pattern (Madagascar, DRC, Peru, Kazakhstan, etc.) or where it is a mostly incidental phenomenon (US, China, Libya, Zambia, Algeria). Furthermore, there are scenarios in which sporadic cases emerge from a contact within a current epizootic focus, or those of a major outbreaks or epidemics. Indeed, plague has a natural tendency to progress, disperse, and eventually, to expand to other regions, as it was observed during the 1990s in Madagascar when plague was established in the city harbour of Mahajanga [99]. This urban focus was sustained by the Asian house shrew (*Suncus murinus*) but transmitted to humans through the black rat (*R*. *rattus*), which exemplifies the surprising adaptability of *Y*. *pestis* [100]. In this sense, it is important to note the nature of *Y*. *pestis* as a “generalist” pathogen, demonstrated by its ability to evolve across different environments, vectors, and hosts, facilitating its capacity to move from one region, context, or scenario to another, or even the emergence or re-emergence in previously free areas or where human plague has not been observed for decades, as it has been already observed in North and East Africa [101–103]. The apparent unpredictability of plague re-occurrence and emergence highlights how much about the eco-epidemiology of plague remains poorly understood, and this should be a matter of great concern for public health officials. Studies during on-going outbreaks may provide important insights about the epidemic dynamics of human plague.

More context-specific research should be framed in a translational strategy in order to produce appropriate tools, devices, and policies. Translational research should be considered in their 2 widely used meanings: the “bench-to-bedside” process—which involves applying knowledge from basic sciences to produce new medicines, diagnostic tools, and treatment options for patients—and from a public health perspective whereby work focuses on healthcare delivery systems to improve health services research and to have a primary outcome of new policies and practices. In the case of plague, new drugs, vaccines, and diagnostic devices are clearly needed.

**Fig 2:**
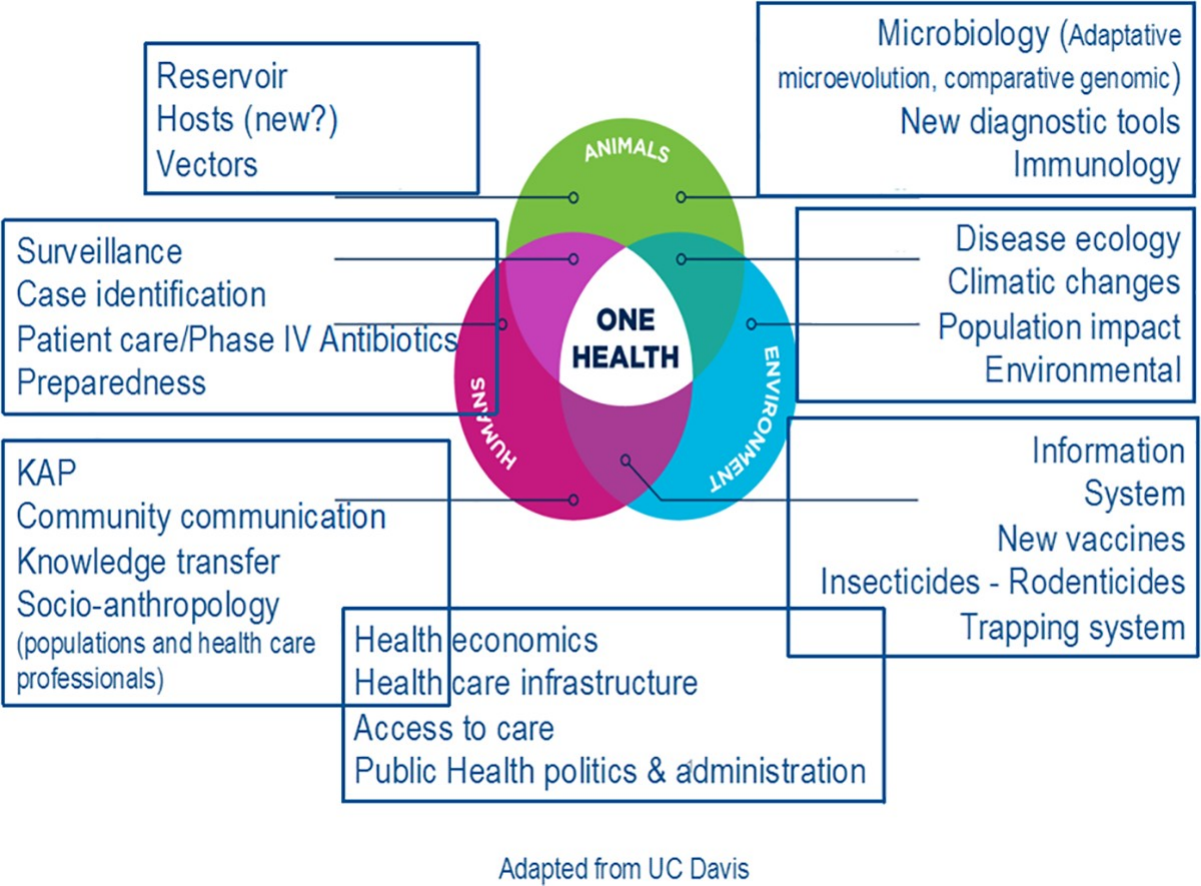
“One Health” integrative approach. KAP, Knowledge, Attitudes and Practices.

Furthermore, more research on the surveillance, ecology, and human behavioural interventions is urgently required. Special efforts should be made to obtain community engagement towards the public health response. To this end, social scientists and anthropologists can provide invaluable insights that can mitigate distrust, increase cooperation, and improve community communication. This cross-cutting research should be linked to the paradigm of One Health (Fig 2), which is grounded in the recognition that human, animal, and environmental health are interdependent. Human plague is a good example of this interdependence where improving human living conditions in plague-endemic areas through environmental and behavioural interventions might be the most effective way to avoid human plague cases as well as reduce environmental degradation to improve ecosystem resilience. The Ebola crisis in West Africa in 2013–2015 shows the capacity of deadly diseases, like human plague, to disrupt societies and health systems [104, 105], as we are experiencing with the current COVID-19 pandemic. Growing urbanization, environmental/climate change, and population mobility increase the chances of such phenomena. In summary, we ascertained the main axes of research that should be prioritized for plague prevention and control: (i) an improved understanding of the ecological interactions among the reservoir, vector, pathogen, and environment; (ii) human and societal responses; (iii) improved diagnostic tools and case management; and (iv) vaccine development.

Accordingly, as a final point highlighted from the Paris workshop, plague research needs an inter-and transdisciplinary approach that involves molecular biologists, immunologists, clinicians, epidemiologists, public health specialists, veterinarians, zoologists, entomologists, mathematical modelling specialists, ecologists, anthropologists, and social scientists. Even in emergency situations, the involvement of anthropologists and social scientists has been considered increasingly relevant [106, 107]. An example of such a cross-cutting research could be the identification of reservoir hosts to be targeted for the development and implementation of wildlife vaccination programmes. As was suggested during the workshop, to make human plague history has 2 complementary meanings: a history with a happy end or to make plague a relic of the past.

**Top five papers**

1. Wu L. League of Nations. A treatise on pneumonic plague. Nancy: Berger-Levrault, 1925.
2. Pollitzer R. Plague studies. VIII. Clinical aspects. Bull World Health Organ. 1953; 9(1):59–129.
3. Denis DT, Gage KL, Gratz N, Poland JD, Tikhomirov E. Plague manual: Epidemiology, Distribution, Surveillance and Control. WHO. 1999. [cited 2020 June 22]. Available from: http://www.who.int/csr/resources/publications/plague/whocdscsredc992a.pdf?ua=1
4. Gage Kl, Kosoy MY. Natural history of plague: perspectives from more than a century of research. Annu Rev Entomol. 2005; 50:505–28.
5. Hinnebusch BJ. The Evolution of flea-borne transmission in *Yersinia pestis*. Curr Issues Mol Biol. 2005; 7:197–212.

**Key learning points**

- The number of human plague cases by current surveillance systems may be underestimated.
- The actual knowledge of vectors and reservoirs implicated in plague transmission and accidental hosts is far from complete.
- The interplay between plague occurrence, human behaviours, climate, and landscape is undeniable.
- New reliable POC diagnostic tests are necessary for both bubonic and pneumonic plague for an efficient outbreak response, especially for pneumonic plague outbreaks.
- The validation of therapeutic alternatives to streptomycin is urgently needed.
- It is necessary to develop a better understanding of human immune responses to plague and to use that information for accelerating vaccine development and validation.
